# Crystal structure of 1,7,8,9-tetra­chloro-4-(2-fluoro­benz­yl)-10,10-dimeth­oxy-4-aza­tri­cyclo­[5.2.1.0^2,6^]dec-8-ene-3,5-dione

**DOI:** 10.1107/S2056989014026279

**Published:** 2015-01-01

**Authors:** Jia-liang Zhong, Jia-wei Hou, Li-hong Liu, He Liu

**Affiliations:** a Shanghai Institute of Pharmaceutical Industry, Shanghai 200040, People’s Republic of China; b Beijing Chao-Yang Hospital, Capital Medical University, Beijing 100020, People’s Republic of China

**Keywords:** crystal structure, biochemical activity, tri­cyclo­[5,2,1,0^2,6^]dec-8-ene-3,5-dione, hydrogen bonding, C—H⋯F inter­action

## Abstract

In the title compound, C_17_H_12_Cl_4_FNO_4_, the configuration of the cyclo­alkene skeleton is *endo,cis*. The benzene ring is twisted by 71.01 (11)° from the attached pyrrolidine ring. In the crystal, one of the methine groups of the fused-ring system forms a weak C—H⋯O hydrogen bond. The other methine groups participates in a C—H⋯F inter­action to the same adjacent mol­ecule. Together, these generate [010] chains.

## Related literature   

For similar structures, see: Shan *et al.* (2012 ▸); Kossakowski *et al.* (2009 ▸). For the biochemical activity of related compounds, see: Kossakowski *et al.* (2006 ▸, 2008 ▸); Struga *et al.* (2007 ▸).
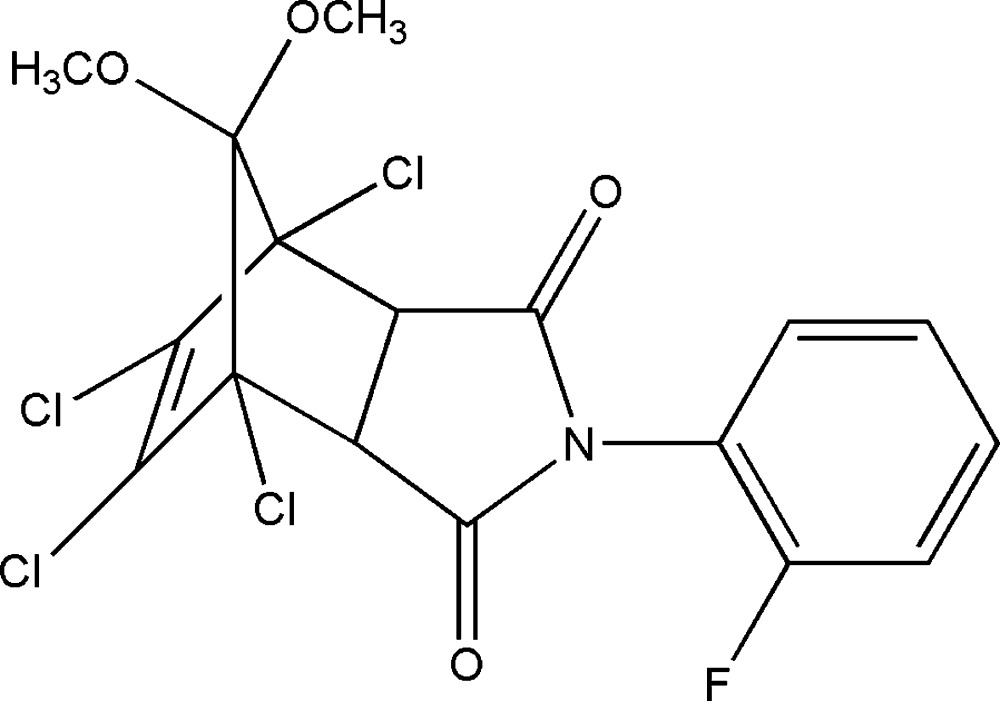


## Experimental   

### Crystal data  

C_17_H_12_Cl_4_FNO_4_
*M* *_r_* = 455.08
Orthorhombic, 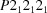

*a* = 9.965 (2) Å
*b* = 10.982 (2) Å
*c* = 16.926 (3) Å
*V* = 1852.1 (6) Å^3^
*Z* = 4
Mo *K*α radiation
μ = 0.67 mm^−1^
*T* = 296 K
0.20 × 0.15 × 0.10 mm

### Data collection  

Bruker APEXII CCD diffractometer
17912 measured reflections
4238 independent reflections
3231 reflections with *I* > 2σ(*I*)
*R*_int_ = 0.069

### Refinement  

*R*[*F* ^2^ > 2σ(*F* ^2^)] = 0.046
*wR*(*F* ^2^) = 0.101
*S* = 1.00
4238 reflections
244 parameters
H-atom parameters constrained
Δρ_max_ = 0.28 e Å^−3^
Δρ_min_ = −0.27 e Å^−3^
Absolute structure: Flack (1983 ▸), 1826 Friedel pairs
Absolute structure parameter: −0.02 (7)

### 

Data collection: *APEX2* (Bruker, 2009 ▸); cell refinement: *SAINT* (Bruker, 2009 ▸); data reduction: *SAINT*; program(s) used to solve structure: *SHELXS97* (Sheldrick, 2008 ▸); program(s) used to refine structure: *SHELXL97* (Sheldrick, 2008 ▸); molecular graphics: *SHELXTL* (Sheldrick, 2008 ▸); software used to prepare material for publication: *SHELXTL*.

## Supplementary Material

Crystal structure: contains datablock(s) I, global. DOI: 10.1107/S2056989014026279/hb7331sup1.cif

Structure factors: contains datablock(s) I. DOI: 10.1107/S2056989014026279/hb7331Isup2.hkl

Supporting information file. DOI: 10.1107/S2056989014026279/hb7331Isup3.cml

**Fig. 1.**
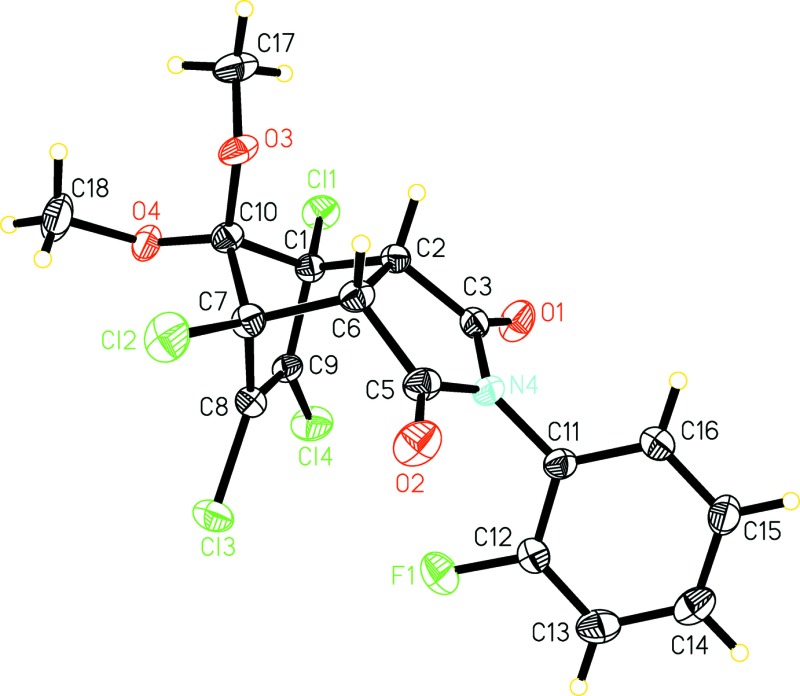
View of the molecule of (I) showing displacement ellipsoids drawn at the 30% probability level.

. DOI: 10.1107/S2056989014026279/hb7331fig1.tif
View of the mol­ecule of (I) showing displacement ellipsoids drawn at the 30% probability level.

**Fig. 2.**
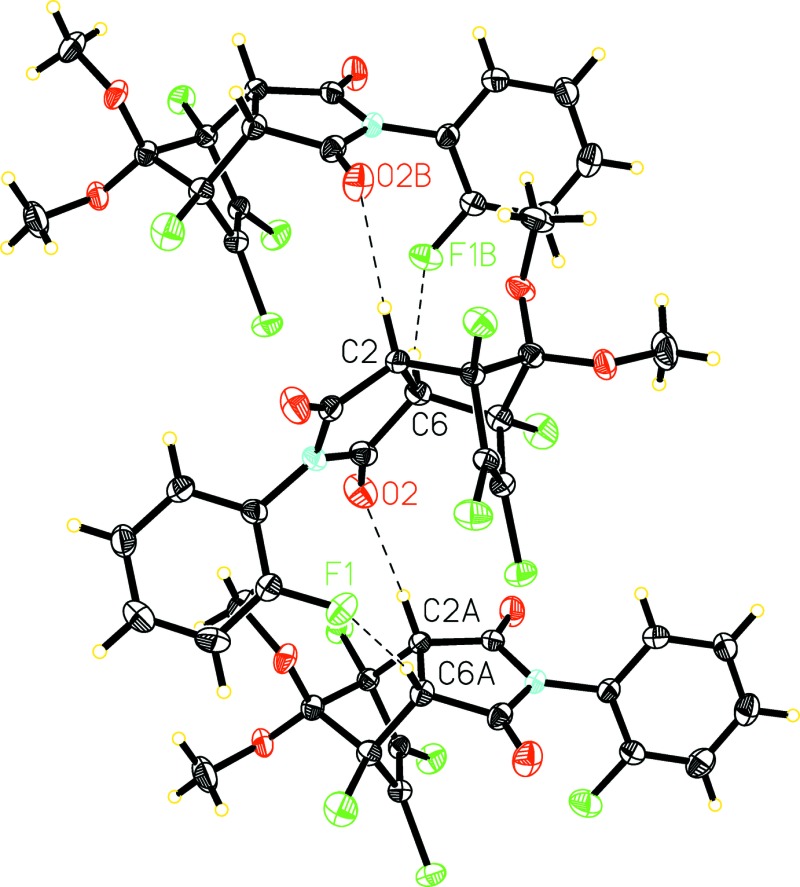
The C—H···X(X=O/F) interactions, dashed lines. Non-essential H atoms are omitted for clarity. Symmetry code: (i) 1 - x, 1/2 + y, 1/2 - z. (ii) 1 - x, y - 1/2, 1/2 - z.

Click here for additional data file.X X x y z x y z . DOI: 10.1107/S2056989014026279/hb7331fig2.tif
The C—H⋯*X*(*X*=O/F) inter­actions, dashed lines. Non-essential H atoms are omitted for clarity. Symmetry code: (i) 1 − *x*, 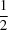 + *y*, 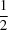 − *z*. (ii) 1 − *x*, *y* − 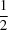, 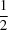 − *z*.

CCDC reference: 1036764

Additional supporting information:  crystallographic information; 3D view; checkCIF report

## Figures and Tables

**Table 1:** Hydrogen-bond geometry (, )

*D*H*A*	*D*H	H*A*	*D* *A*	*D*H*A*
C2H2*A*O2^i^	0.98	2.55	3.487(4)	159
C6H6*A*F1^i^	0.98	2.53	3.500(4)	170

Symmetry code: (i) 
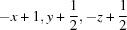
.

## supplementary crystallographic information

### S1. Comment

The title compound, (I)(Fig.1), 1,7,8,9-tetrachloro-4-(2'-fluorobenzyl) -10,10-dimethoxy-4-azatricyclo(5,2,1,0^2,6^)dec-8-ene-3,5-dione was synthesized from *N*-(2'-fluorobenzyl)maleimide and 5,5-dimethoxy- 1,2,3,4-tetrachlorocydopentadiene.

The fused pyrrolidine ring systems, are frequently encountered structural units in many synthetically challenging and biologically active alkaloids. The interest of constructing skeletons of this type was further enlightened by the recent disclosure of Kossakowski *et al.*, (2006) that the rigid arylcyclo analogues having azatricyclo ring systems show anti-HIV-1, anti-cancer, antiviral, and antibacterial activities. We have synthesized a serial compounds with this cycloalkene skeleton. This report is one of them.

In the crystal structure, there is a tricyclic fused pyrrolidine ring system. The configuration of the cycloalkene skeleton is *endo, cis*. The dihedral angle of pyrrolidine ring and benzene ring is 71.01 (11)°.

The molecules packed in spacegroup *P*2_1_2_1_2_1_, and the absolute configuration of the title compound can be determined from Flack parameter *x*=-0.02 (7), and the compound has chirality at C1S, C2S, C6*R*, C7*R*.

Weak intermolecular C—H···*X*(*X*=O,F) hydrogen bonds can be found between adjacent molecules. In details(Table 1), C2—H2A and C6—H6A of the same molecule(1 - *x*, 1/2 + *y*, 1/2 - *z*) provide H-bonds donors to O2, F1, respectively. These pairs of H-bonds link the neighbour molecules along *b*axis to form infinite chains. Another two molecules in the unit cell along *b*axis linked by the same weak H-bonds in the opposite direction. So the whole crystal packing exists as countless helices along *b*axis.

### S2. Experimental

The synthetic pathway for the title compound is described as follows. *N*-(2'fluorobenzyl)maleimide (1.9 g, 10 mmol) and 5,5- dimethoxy- 1,2,3,4-tetrachlorocydopentadiene (2.63 g, 10 mmol) were dissolved in anhydrous toluene (100 mL). Then the solution was refluxed for 8 h. After the solvent was removed under reduced pressure, the residue was dissolved in ether (150 mL), washed with water and brine, dried over anhydrous sodium sulfate, and concentrated to dryness. The product was purified by flash-chromatography (petroleum ether/ethyl acetate, 6:1) and the title compound was isolated as a white solid (3.86 g, 85%). m.p.: 116–118°C.

The crystals appropriate for X-ray data collection were obtained from ethyl acetate solution at room temperature after two days.

### S3. Refinement

All H atoms were placed in geometically idealized positions and constrained to ride on their parent atoms with C—H distances of 0.93 Å (0.98 for alicylic CH) for aromatic ring CH, and *U*_iso_(H) = 1.2(1.5 for CH_3_)*U*_eq_(C).

### Crystal data

**Table:**

C_17_H_12_Cl_4_FNO_4_	*F*(000) = 920
*M**_r_* = 455.08	*D*_x_ = 1.632 Mg m^−^^3^
Orthorhombic, *P*2_1_2_1_2_1_	Mo *K*α radiation, λ = 0.71073 Å
Hall symbol: P 2ac 2ab	Cell parameters from 4520 reflections
*a* = 9.965 (2) Å	θ = 3.0–27.5°
*b* = 10.982 (2) Å	µ = 0.67 mm^−^^1^
*c* = 16.926 (3) Å	*T* = 296 K
*V* = 1852.1 (6) Å^3^	Prismatic, colorless
*Z* = 4	0.20 × 0.15 × 0.10 mm

### Data collection

**Table:**

Bruker APEXII CCD diffractometer	3231 reflections with *I* > 2σ(*I*)
Radiation source: fine-focus sealed tube	*R*_int_ = 0.069
Graphite monochromator	θ_max_ = 27.5°, θ_min_ = 3.0°
φ and ω scans	*h* = −12→12
17912 measured reflections	*k* = −14→14
4238 independent reflections	*l* = −21→21

### Refinement

**Table:**

Refinement on *F*^2^	Secondary atom site location: difference Fourier map
Least-squares matrix: full	Hydrogen site location: inferred from neighbouring sites
*R*[*F*^2^ > 2σ(*F*^2^)] = 0.046	H-atom parameters constrained
*wR*(*F*^2^) = 0.101	*w* = 1/[σ^2^(*F*_o_^2^) + (0.050*P*)^2^] where *P* = (*F*_o_^2^ + 2*F*_c_^2^)/3
*S* = 1.00	(Δ/σ)_max_ < 0.001
4238 reflections	Δρ_max_ = 0.28 e Å^−^^3^
244 parameters	Δρ_min_ = −0.27 e Å^−^^3^
0 restraints	Absolute structure: Flack (1983), 1826 Friedel pairs
Primary atom site location: structure-invariant direct methods	Absolute structure parameter: −0.02 (7)

### Special details

**Table:**

Geometry. All e.s.d.'s (except the e.s.d. in the dihedral angle between two l.s. planes) are estimated using the full covariance matrix. The cell e.s.d.'s are taken into account individually in the estimation of e.s.d.'s in distances, angles and torsion angles; correlations between e.s.d.'s in cell parameters are only used when they are defined by crystal symmetry. An approximate (isotropic) treatment of cell e.s.d.'s is used for estimating e.s.d.'s involving l.s. planes.
Refinement. Refinement of *F*^2^ against ALL reflections. The weighted *R*-factor *wR* and goodness of fit *S* are based on *F*^2^, conventional *R*-factors *R* are based on *F*, with *F* set to zero for negative *F*^2^. The threshold expression of *F*^2^ > σ(*F*^2^) is used only for calculating *R*-factors(gt) *etc*. and is not relevant to the choice of reflections for refinement. *R*-factors based on *F*^2^ are statistically about twice as large as those based on *F*, and *R*- factors based on ALL data will be even larger.

### Fractional atomic coordinates and isotropic or equivalent isotropic displacement parameters (Å^2^)

**Table:**

	*x*	*y*	*z*	*U*_iso_*/*U*_eq_	
Cl1	0.09057 (8)	0.72603 (7)	0.11428 (5)	0.0470 (2)	
Cl2	0.31474 (10)	0.49911 (9)	0.37871 (5)	0.0614 (3)	
Cl3	0.28576 (9)	0.30235 (7)	0.23337 (6)	0.0541 (2)	
Cl4	0.16447 (10)	0.44681 (8)	0.07087 (5)	0.0549 (3)	
F1	0.5234 (2)	0.33581 (19)	0.09916 (14)	0.0675 (6)	
O1	0.4175 (2)	0.6801 (2)	0.03909 (14)	0.0582 (7)	
O2	0.5994 (2)	0.4890 (2)	0.25327 (14)	0.0578 (6)	
O3	0.1973 (2)	0.74679 (18)	0.30831 (13)	0.0420 (5)	
O4	0.0408 (2)	0.59723 (19)	0.27636 (13)	0.0416 (5)	
N4	0.5329 (2)	0.5781 (2)	0.13621 (15)	0.0354 (6)	
C1	0.2012 (3)	0.6451 (2)	0.17531 (17)	0.0305 (6)	
C2	0.3479 (3)	0.6939 (3)	0.17719 (17)	0.0328 (6)	
H2A	0.3501	0.7825	0.1839	0.039*	
C3	0.4315 (3)	0.6544 (3)	0.1073 (2)	0.0380 (7)	
C5	0.5247 (3)	0.5554 (3)	0.21679 (19)	0.0394 (7)	
C6	0.4076 (3)	0.6283 (3)	0.24928 (18)	0.0354 (7)	
H6A	0.4383	0.6870	0.2889	0.042*	
C7	0.2890 (3)	0.5511 (3)	0.28159 (17)	0.0365 (7)	
C8	0.2570 (3)	0.4540 (3)	0.22159 (18)	0.0347 (7)	
C9	0.2065 (3)	0.5094 (3)	0.15910 (17)	0.0335 (6)	
C10	0.1705 (3)	0.6410 (3)	0.26658 (18)	0.0344 (7)	
C11	0.6311 (3)	0.5241 (3)	0.08502 (18)	0.0357 (7)	
C12	0.6251 (3)	0.4016 (3)	0.06730 (19)	0.0400 (7)	
C13	0.7166 (4)	0.3465 (3)	0.0191 (2)	0.0529 (9)	
H13A	0.7117	0.2634	0.0090	0.063*	
C14	0.8162 (4)	0.4165 (3)	−0.0142 (2)	0.0517 (9)	
H14A	0.8782	0.3810	−0.0481	0.062*	
C15	0.8242 (3)	0.5388 (3)	0.0026 (2)	0.0511 (9)	
H15A	0.8925	0.5855	−0.0195	0.061*	
C16	0.7320 (3)	0.5925 (3)	0.0517 (2)	0.0458 (8)	
H16A	0.7378	0.6753	0.0626	0.055*	
C17	0.1104 (3)	0.8490 (3)	0.2942 (2)	0.0523 (9)	
H17A	0.1389	0.9166	0.3260	0.078*	
H17B	0.0199	0.8275	0.3078	0.078*	
H17C	0.1144	0.8712	0.2394	0.078*	
C18	−0.0074 (4)	0.5857 (4)	0.3562 (2)	0.0682 (12)	
H18A	−0.0975	0.5547	0.3556	0.102*	
H18B	−0.0063	0.6640	0.3814	0.102*	
H18C	0.0493	0.5306	0.3848	0.102*	

### Atomic displacement parameters (Å^2^)

**Table:**

	*U*^11^	*U*^22^	*U*^33^	*U*^12^	*U*^13^	*U*^23^
Cl1	0.0449 (4)	0.0488 (4)	0.0472 (5)	0.0125 (4)	−0.0076 (4)	0.0056 (4)
Cl2	0.0770 (6)	0.0779 (6)	0.0295 (4)	0.0107 (5)	−0.0045 (4)	0.0089 (4)
Cl3	0.0587 (5)	0.0322 (4)	0.0714 (6)	0.0060 (4)	0.0034 (5)	0.0080 (4)
Cl4	0.0725 (6)	0.0539 (5)	0.0382 (5)	0.0019 (4)	−0.0065 (4)	−0.0174 (4)
F1	0.0668 (13)	0.0489 (11)	0.0869 (17)	−0.0211 (11)	0.0124 (13)	−0.0032 (11)
O1	0.0571 (15)	0.0707 (17)	0.0467 (15)	0.0238 (14)	0.0139 (13)	0.0245 (13)
O2	0.0512 (13)	0.0732 (16)	0.0489 (14)	0.0201 (14)	−0.0076 (12)	0.0059 (13)
O3	0.0397 (12)	0.0425 (12)	0.0438 (12)	0.0047 (10)	0.0007 (10)	−0.0187 (10)
O4	0.0354 (12)	0.0516 (13)	0.0380 (13)	−0.0048 (10)	0.0104 (9)	−0.0076 (10)
N4	0.0318 (13)	0.0332 (13)	0.0410 (16)	0.0069 (11)	0.0050 (11)	0.0015 (12)
C1	0.0289 (15)	0.0310 (14)	0.0315 (15)	0.0016 (12)	−0.0015 (13)	−0.0001 (12)
C2	0.0293 (14)	0.0277 (14)	0.0413 (17)	0.0005 (12)	0.0025 (13)	−0.0005 (13)
C3	0.0356 (16)	0.0309 (15)	0.048 (2)	0.0011 (13)	0.0073 (15)	0.0087 (14)
C5	0.0314 (16)	0.0379 (16)	0.049 (2)	0.0036 (14)	−0.0049 (15)	−0.0054 (15)
C6	0.0324 (15)	0.0363 (15)	0.0374 (17)	0.0006 (13)	−0.0038 (14)	−0.0072 (14)
C7	0.0409 (16)	0.0403 (15)	0.0282 (16)	0.0041 (14)	−0.0019 (14)	0.0023 (13)
C8	0.0369 (16)	0.0315 (14)	0.0356 (16)	0.0003 (13)	0.0015 (14)	0.0023 (13)
C9	0.0369 (15)	0.0328 (14)	0.0308 (15)	0.0001 (14)	0.0031 (12)	−0.0060 (13)
C10	0.0360 (16)	0.0357 (15)	0.0316 (16)	0.0008 (13)	−0.0004 (13)	−0.0054 (12)
C11	0.0343 (15)	0.0340 (15)	0.0389 (16)	0.0052 (13)	0.0005 (13)	0.0005 (13)
C12	0.0393 (18)	0.0385 (17)	0.0422 (19)	−0.0049 (14)	0.0008 (14)	0.0015 (14)
C13	0.062 (2)	0.0429 (18)	0.054 (2)	0.0043 (18)	−0.0030 (19)	−0.0170 (17)
C14	0.052 (2)	0.063 (2)	0.0401 (19)	0.0176 (18)	0.0028 (17)	−0.0024 (17)
C15	0.0406 (19)	0.055 (2)	0.057 (2)	0.0036 (17)	0.0133 (18)	0.0102 (18)
C16	0.0453 (19)	0.0336 (16)	0.058 (2)	−0.0006 (15)	0.0057 (17)	0.0055 (16)
C17	0.047 (2)	0.0437 (18)	0.066 (2)	0.0124 (16)	−0.0026 (18)	−0.0189 (17)
C18	0.064 (3)	0.086 (3)	0.054 (2)	−0.009 (2)	0.030 (2)	−0.004 (2)

### Geometric parameters (Å, º)

**Table:**

Cl1—C1	1.753 (3)	C6—C7	1.554 (4)
Cl2—C7	1.759 (3)	C6—H6A	0.9800
Cl3—C8	1.702 (3)	C7—C8	1.506 (4)
Cl4—C9	1.696 (3)	C7—C10	1.560 (4)
F1—C12	1.356 (4)	C8—C9	1.320 (4)
O1—C3	1.197 (4)	C11—C16	1.376 (4)
O2—C5	1.211 (4)	C11—C12	1.380 (4)
O3—C10	1.385 (3)	C12—C13	1.365 (5)
O3—C17	1.438 (4)	C13—C14	1.376 (5)
O4—C10	1.389 (3)	C13—H13A	0.9300
O4—C18	1.440 (4)	C14—C15	1.375 (5)
N4—C5	1.389 (4)	C14—H14A	0.9300
N4—C3	1.401 (4)	C15—C16	1.372 (5)
N4—C11	1.435 (4)	C15—H15A	0.9300
C1—C9	1.516 (4)	C16—H16A	0.9300
C1—C2	1.557 (4)	C17—H17A	0.9600
C1—C10	1.576 (4)	C17—H17B	0.9600
C2—C3	1.511 (4)	C17—H17C	0.9600
C2—C6	1.537 (4)	C18—H18A	0.9600
C2—H2A	0.9800	C18—H18B	0.9600
C5—C6	1.518 (4)	C18—H18C	0.9600
			
C10—O3—C17	117.0 (2)	C8—C9—Cl4	127.8 (2)
C10—O4—C18	116.9 (3)	C1—C9—Cl4	123.3 (2)
C5—N4—C3	114.1 (3)	O3—C10—O4	114.1 (2)
C5—N4—C11	124.0 (2)	O3—C10—C7	107.6 (2)
C3—N4—C11	121.9 (3)	O4—C10—C7	117.7 (2)
C9—C1—C2	108.0 (2)	O3—C10—C1	116.0 (2)
C9—C1—C10	99.0 (2)	O4—C10—C1	107.9 (2)
C2—C1—C10	99.9 (2)	C7—C10—C1	91.8 (2)
C9—C1—Cl1	114.4 (2)	C16—C11—C12	118.4 (3)
C2—C1—Cl1	115.35 (19)	C16—C11—N4	121.3 (3)
C10—C1—Cl1	118.0 (2)	C12—C11—N4	120.3 (3)
C3—C2—C6	105.9 (2)	F1—C12—C13	120.0 (3)
C3—C2—C1	113.7 (2)	F1—C12—C11	117.7 (3)
C6—C2—C1	102.6 (2)	C13—C12—C11	122.2 (3)
C3—C2—H2A	111.4	C12—C13—C14	118.6 (3)
C6—C2—H2A	111.4	C12—C13—H13A	120.7
C1—C2—H2A	111.4	C14—C13—H13A	120.7
O1—C3—N4	124.2 (3)	C15—C14—C13	120.2 (3)
O1—C3—C2	128.5 (3)	C15—C14—H14A	119.9
N4—C3—C2	107.2 (3)	C13—C14—H14A	119.9
O2—C5—N4	124.9 (3)	C16—C15—C14	120.3 (3)
O2—C5—C6	127.2 (3)	C16—C15—H15A	119.8
N4—C5—C6	107.8 (2)	C14—C15—H15A	119.8
C5—C6—C2	104.9 (2)	C15—C16—C11	120.2 (3)
C5—C6—C7	115.1 (2)	C15—C16—H16A	119.9
C2—C6—C7	104.0 (2)	C11—C16—H16A	119.9
C5—C6—H6A	110.8	O3—C17—H17A	109.5
C2—C6—H6A	110.8	O3—C17—H17B	109.5
C7—C6—H6A	110.8	H17A—C17—H17B	109.5
C8—C7—C6	108.0 (2)	O3—C17—H17C	109.5
C8—C7—C10	100.3 (2)	H17A—C17—H17C	109.5
C6—C7—C10	99.9 (2)	H17B—C17—H17C	109.5
C8—C7—Cl2	115.6 (2)	O4—C18—H18A	109.5
C6—C7—Cl2	113.3 (2)	O4—C18—H18B	109.5
C10—C7—Cl2	117.9 (2)	H18A—C18—H18B	109.5
C9—C8—C7	107.1 (2)	O4—C18—H18C	109.5
C9—C8—Cl3	127.5 (2)	H18A—C18—H18C	109.5
C7—C8—Cl3	125.3 (2)	H18B—C18—H18C	109.5
C8—C9—C1	108.7 (3)		
			
C9—C1—C2—C3	49.3 (3)	Cl1—C1—C9—C8	−160.5 (2)
C10—C1—C2—C3	152.2 (2)	C2—C1—C9—Cl4	−106.0 (3)
Cl1—C1—C2—C3	−80.2 (3)	C10—C1—C9—Cl4	150.4 (2)
C9—C1—C2—C6	−64.6 (3)	Cl1—C1—C9—Cl4	24.0 (3)
C10—C1—C2—C6	38.3 (2)	C17—O3—C10—O4	−55.9 (4)
Cl1—C1—C2—C6	165.98 (19)	C17—O3—C10—C7	171.5 (3)
C5—N4—C3—O1	−176.4 (3)	C17—O3—C10—C1	70.5 (3)
C11—N4—C3—O1	0.4 (5)	C18—O4—C10—O3	−49.8 (4)
C5—N4—C3—C2	3.3 (3)	C18—O4—C10—C7	77.8 (4)
C11—N4—C3—C2	−179.9 (3)	C18—O4—C10—C1	179.7 (3)
C6—C2—C3—O1	177.2 (3)	C8—C7—C10—O3	−170.1 (2)
C1—C2—C3—O1	65.3 (4)	C6—C7—C10—O3	−59.5 (3)
C6—C2—C3—N4	−2.4 (3)	Cl2—C7—C10—O3	63.6 (3)
C1—C2—C3—N4	−114.3 (3)	C8—C7—C10—O4	59.3 (3)
C3—N4—C5—O2	177.5 (3)	C6—C7—C10—O4	169.9 (2)
C11—N4—C5—O2	0.8 (5)	Cl2—C7—C10—O4	−67.0 (3)
C3—N4—C5—C6	−2.7 (3)	C8—C7—C10—C1	−52.0 (2)
C11—N4—C5—C6	−179.4 (3)	C6—C7—C10—C1	58.5 (2)
O2—C5—C6—C2	−179.2 (3)	Cl2—C7—C10—C1	−178.3 (2)
N4—C5—C6—C2	0.9 (3)	C9—C1—C10—O3	161.4 (2)
O2—C5—C6—C7	−65.6 (4)	C2—C1—C10—O3	51.1 (3)
N4—C5—C6—C7	114.5 (3)	Cl1—C1—C10—O3	−74.7 (3)
C3—C2—C6—C5	0.9 (3)	C9—C1—C10—O4	−69.1 (3)
C1—C2—C6—C5	120.4 (2)	C2—C1—C10—O4	−179.4 (2)
C3—C2—C6—C7	−120.4 (3)	Cl1—C1—C10—O4	54.8 (3)
C1—C2—C6—C7	−0.9 (3)	C9—C1—C10—C7	50.8 (2)
C5—C6—C7—C8	−47.1 (3)	C2—C1—C10—C7	−59.4 (2)
C2—C6—C7—C8	67.1 (3)	Cl1—C1—C10—C7	174.7 (2)
C5—C6—C7—C10	−151.4 (2)	C5—N4—C11—C16	−110.9 (4)
C2—C6—C7—C10	−37.3 (3)	C3—N4—C11—C16	72.6 (4)
C5—C6—C7—Cl2	82.2 (3)	C5—N4—C11—C12	69.9 (4)
C2—C6—C7—Cl2	−163.61 (19)	C3—N4—C11—C12	−106.6 (3)
C6—C7—C8—C9	−68.4 (3)	C16—C11—C12—F1	−178.6 (3)
C10—C7—C8—C9	35.7 (3)	N4—C11—C12—F1	0.6 (5)
Cl2—C7—C8—C9	163.6 (2)	C16—C11—C12—C13	1.0 (5)
C6—C7—C8—Cl3	110.2 (3)	N4—C11—C12—C13	−179.8 (3)
C10—C7—C8—Cl3	−145.7 (2)	F1—C12—C13—C14	178.1 (3)
Cl2—C7—C8—Cl3	−17.8 (3)	C11—C12—C13—C14	−1.5 (5)
C7—C8—C9—C1	−0.7 (3)	C12—C13—C14—C15	1.4 (6)
Cl3—C8—C9—C1	−179.2 (2)	C13—C14—C15—C16	−0.9 (6)
C7—C8—C9—Cl4	174.6 (2)	C14—C15—C16—C11	0.3 (5)
Cl3—C8—C9—Cl4	−4.0 (4)	C12—C11—C16—C15	−0.4 (5)
C2—C1—C9—C8	69.5 (3)	N4—C11—C16—C15	−179.6 (3)
C10—C1—C9—C8	−34.1 (3)		

### Hydrogen-bond geometry (Å, º)

**Table:**

*D*—H···*A*	*D*—H	H···*A*	*D*···*A*	*D*—H···*A*
C2—H2*A*···O2^i^	0.98	2.55	3.487 (4)	159
C6—H6*A*···F1^i^	0.98	2.53	3.500 (4)	170

Symmetry code: (i) −*x*+1, *y*+1/2, −*z*+1/2.
